# Effect of Midface Surgery on Ocular Outcomes in Patients with Orbital and Midface Malformations

**DOI:** 10.3390/jcm12113862

**Published:** 2023-06-05

**Authors:** Parinaz Rostamzad, Mieke M. Pleumeekers, Sarah L. Versnel, Sjoukje E. Loudon

**Affiliations:** 1 Department of Plastic and Reconstructive Surgery, Erasmus Medical Center, 3000 CA Rotterdam, The Netherlands; 2 Department of Ophthalmology, Erasmus Medical Center, 3000 CA Rotterdam, The Netherlands

**Keywords:** midface surgery, maxillofacial surgery, peri-orbital malformations, hypertelorism, midface hypoplasia, orbital dysplasia, craniofacial disorders

## Abstract

(1) Background: Orbital and midface malformations occur in multiple craniofacial disorders. Depending on the deformity, surgical corrections include orbital box osteotomy (OBO), Le Fort III (LFIII), monobloc (MB), and facial bipartition (FB). The aim of this study was to determine the effect of these procedures on ocular outcomes. (2) Methods: A retrospective analysis was performed. All patients with craniofacial disorders who had previously undergone midface surgery were included. The Wilcoxon signed ranks test was used for statistical analysis. (3) Results: In total, 63 patients were included: two patients were treated by OBO, 20 by LFIII, 26 by MB, and 15 by FB. Pre-operatively, strabismus was present in 39 patients (61.9%), in whom exotropia was most common (*n* = 27; 42.9%), followed by esotropia (*n* = 11; 17.5%). Postoperatively, strabismus significantly worsened (*p* = 0.035) in the overall population (*n* = 63). Pre-operative binocular vision (*n* = 33) was absent in nine patients (27.3%), poor in eight (24.2%), moderate in 15 (45.5%), and good in one (3.0%). Postoperatively, binocular vision significantly improved (*p* < 0.001). Before surgery, the mean visual acuity (VA) in the better eye was 0.16 LogMAR (Logarithm of the Minimum Angle of Resolution), and 0.31 LogMAR in the worse eye. Furthermore, pre-operative astigmatism was present in 46 patients (73.0%) and hypermetropia in 37 patients (58.7%). No statistical difference was found for VA (*n* = 51; *p* = 0.058) postoperatively. (4) Conclusions: Midface surgery has a direct and indirect substantial effect on several ocular outcomes. This study emphasizes the importance of appropriate ophthalmological evaluation in patients with craniofacial disorders undergoing midface surgery.

## 1. Introduction

Orbital and midface malformations occur in multiple congenital craniofacial disorders; for example, craniosynostosis, facial clefts, and craniofrontonasal dysplasia (CFNS). These orbital malformations in craniofacial disorders include hypertelorism (significantly increased interorbital distance), orbital dystopia (abnormal displacement of the orbit and its contents), and midface hypoplasia (underdevelopment of the midface) [1,2]. Patients with these orbital malformations present with a variety of features, including proptosis leading to incomplete closure of the eyelids and exposure keratitis, the inability to develop binocular vision, eye motility disorders, refractive errors, and a decrease in visual acuity (VA) [3]. Consequently, in some cases, surgical correction of the midface and the orbits is required. Depending on the deformity and the anatomical structures involved, surgical techniques include: orbital box osteotomy (OBO), Le Fort III (LFIII), monobloc advancement (MB), or facial bipartition (FB). The OBO, is performed to correct hypertelorism, when midface hypoplasia is absent and normal occlusion is present or in cases of a vertical orbital dystopia [4]. The LFIII is currently performed to advance the midface and zygomas, and to correct the nose [5,6,7]. Furthermore, it is applied for the improvement of malocclusion and the enlargement of the upper airway [7]. In more severe cases, a MB is indicated for enlargement of the skull volume to reduce high intracranial pressure, midface advancement, improvement of proptosis, upper airway obstruction, and malocclusion [8]. Finally, FB is applied to correct hypertelorism and if functional correction of V-shaped malocclusion is needed [9,10]. Although these surgical procedures have been applied for many years, little literature is available on ocular and orbital outcomes, and outcomes vary due to the low number of patients. Previously, it has been stated that the eyes are significantly repositioned after midface surgery, and it appears that the optic nerve and ocular muscles easily adapt to their new position [11,12]. However, worsened strabismus has also been reported after midface surgery [13]. Therefore, this study aimed to determine the effect of the various midface surgeries on short-term ocular outcomes in patients with orbital and midface malformations. This could aid in the decision-making and timing of surgery for the various pathologies in these children.

## 2. Materials and Methods

### 2.1. Study Design and Participants

A retrospective case series was performed on syndromal craniofacial patients who were treated between 2000–2023 at the Sophia Children’s Hospital in Rotterdam, The Netherlands. Patients were included if they had been diagnosed with a type of syndromal craniofacial disorder, consisting of Apert syndrome, Crouzon syndrome, or CFNS who underwent surgical correction of the midface and/or orbita, consisting of OBO, LFIII, MB, and FB. Furthermore, patients were included if genetic testing of syndromal craniofacial disorders was confirmed, and if pre- and postoperative ophthalmological examinations were available. Patients were included if the pre-operative ophthalmological examination was up to 6 months before surgery, and if postoperative examinations were available between 3–12 months. This range of periods was used to reduce the chance that influences other than midface surgery would have an impact on the ocular outcomes. In addition, midface surgery was often combined with distraction osteogenesis, therefore ocular examinations were included after the removal of the distractors. Patients were excluded if a genetic diagnosis was not available or if patients were diagnosed as non-syndromal craniosynostosis or other types of craniofacial disorders. Patients were excluded if pre- or postoperative ophthalmological examinations were missing or not in the range of the inclusion period. Patients were excluded if they underwent other types of surgical correction of the midface than OBO, LFIII, MB, and FB. This retrospective study was approved by the Medical Ethical Committee (MEC-2022-0309).

### 2.2. Ocular Anomalies and Orbital Malformations

The following ocular anomalies were included: strabismus (horizontal, vertical, and pattern deviations), decrease in visual acuity (VA), refractive errors (anisometropia, hypermetropia, myopia, and astigmatism), binocular vision, diplopia, amblyopia lacrimal system dysfunction, and papilledema. Orbital measurements consisting of anterior interorbital distance (AIOD) and globe protrusion (GP) were measured on pre-operative (0–6 months) and postoperative (6–12 months) CT scans on the axial plane using Proplan software. Intra-rater reliability for orbital measurements was measured in SPSS using the intraclass correlation coefficient (ICC). An example of the orbital measurements is presented in Supplementary Material A.

### 2.3. Ophthalmological and Orthoptical Examination

The orbital anomalies in patients with craniofacial disorders were initially assessed based on their radiology reports, clinical examinations, and photographs, which were extracted from their electronic medical records. Both orthoptic and ophthalmological examinations were analyzed. The patients within each surgical group were divided into three age groups (0–6 years, 7–12 years, ≥13 years) in order to find any differences within ocular outcomes at different age groups. The cover-uncover test was performed to detect ocular deviations [14,15]. Depending on their age, VA was measured with the Amsterdam Picture Chart (including eleven different optotypes used at 36 months) [16], tumbling E-chart, or Snellen chart [17]. The measurement of VA was converted to LogMAR (Logarithm of the Minimum Angle of Resolution). Refraction was examined in cycloplegia (1% cyclopentolate eye drops). Anisometropia, astigmatism, and hypermetropia were defined as ≥1.00 dioptres and myopia as ≤−1.00 dioptres. An increase or decrease of at least 1 dioptre was indicated as an improvement or deterioration of refractive errors. Depending on their age, binocular vision was measured with Bagolini glasses, Lang-stereotest II, Titmus Fly test, and/or TNO test. Binocular vision was divided into four groups [18]: (1) negative Bagolini was considered as no binocular vision present; (2) positive Bagolini and positive housefly were considered as poor binocular vision; (3) recognition of Titmus circles 200″-140″, and 100″-40″ were considered as moderate binocular vision; and (4) recognition of TNO plate V 480″-240″, TNO plate VI, or VII 120″-15″ were considered as good binocular vision. Funduscopy was used to assess the retina. Papilledema was graded in four groups based on the Frisen staging system [19].

### 2.4. Statistical Analysis

Descriptive statistics were used for both the ocular outcomes and for patient characteristics. All ocular outcomes were separately described for each diagnosis and type of surgery. The Wilcoxon signed ranks test was used in software program SPSS version 28.0 to determine statistical differences between the pre- and postoperative ocular outcomes (strabismus, refractive errors, binocular vision, VA and papilledema) considering the total population together, where no distinction was made between the type of diagnosis or age group, as otherwise the groups for statistical analysis would be too small.

## 3. Results

### 3.1. Study Characteristics

The study characteristics are demonstrated in Table 1. The total study population consisted of 63 patients. The mean age at surgery was 9.4 years (range 1–22.3 years). In total, two patients underwent an OBO, 20 patients a LFIII, 26 patients MB, and 15 patients a FB. The most common surgical indications were malocclusion in 43 patients (68.3%), proptosis with an inability to close the eyelids in 19 patients (30.2%), and obstructive sleep apnea (OSAS) in 22 patients (34.9%). The intra-observer reliability showed a value of 0.92, demonstrating excellent reliability for the orbital measurements on CT scans.

### 3.2. Ophthalmological Examinations

#### 3.2.1. Pre-Operative Measurements

The pre-operative ophthalmological examinations are presented in Table 2. Before surgery, strabismus was present in 39 patients (61.9%), in whom exotropia was most common (*n* = 27; 42.9%), followed by esotropia (*n* = 11; 17.5%). The mean VA in the better eye was 0.16 LogMAR, and 0.31 LogMAR in the worse eye. Furthermore, astigmatism was present in 45 patients (71.4%) and hypermetropia in 43 patients (68.3%). Pre-operative binocular vision—examined in 33 patients—was absent in nine patients (27.3%), poor in eight (24.2%), moderate in 15 (45.5%), and good in one patient (3.0%). Detailed patient characteristics and complete pre- and postoperative ophthalmological examinations of each patient are separately described in Supplementary Material B.

#### 3.2.2. Postoperative Measurements

**Figure 1:**
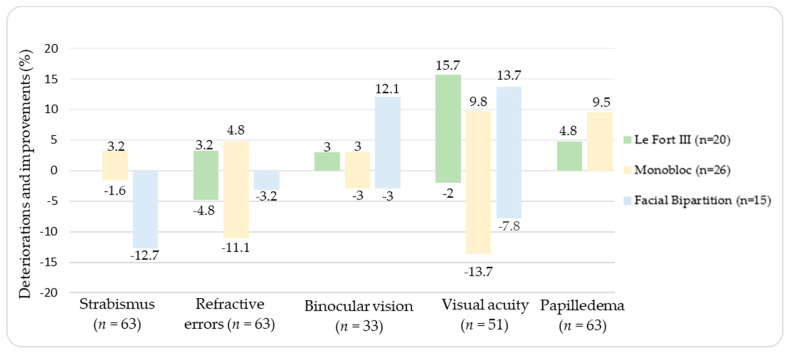
Deteriorations and improvements after midface surgery. PM: note that binocular vision (*n* = 33) and visual acuity (*n* = 51) were not examined in all patients (*n* = 63).

Ocular improvements and deterioration after midface surgery are demonstrated in Table 3 and Table 4 and Figure 1. The mean time for postoperative ophthalmic evaluation was 8 months (±4 months). Strabismus significantly worsened after surgery (*p* = 0.035, z = −2.111) in the overall population (*n* = 63). Pre- and postoperative measurements of VA were available in 51 patients. No statistical difference for VA (*p* = 0.058, z = −1.896) was postoperatively found. There was a significant improvement in binocular vision after midface surgery in patients having a measurement of binocular vision (*p* < 0.001, z = −7.900; *n* = 33). Refractive errors significantly postoperatively worsened in the overall population, (*p* = 0.05, z = −1.964; *n* = 63). Papilledema significantly postoperatively improved in the overall population (*p* < 0.001, z = −4.000; *n* = 63). In addition, five patients postoperatively developed diplopia (LFIII (*n* = 2), MB (*n* = 2), FB (*n* = 1)), although all were resolved within one month without additional treatment. Moreover, two patients had corneal ulceration and keratitis after MB.

Depending on the surgical procedure and craniofacial malformation, strabismus worsened more often after FB in patients with CFNS (50%; *n* = 12). However, patients with CFNS also had more improvement in their binocular vision after FB compared to the other procedures (33.3%; *n* = 12). VA improved most in patients with Crouzon syndrome after LFIII (18.8%; *n* = 32). VA worsened most in patients with Apert syndrome (25%; *n* = 16), followed by Crouzon syndrome (9.4%; *n* = 32) after MB. Papilledema improved most after MB in patients with Crouzon syndrome (12.5%; *n* = 32) and Apert syndrome (18.8%; *n* = 16). No deteriorations were postoperatively found for papilledema.

## 4. Discussion

This study is the first to report on the effect of various surgical corrections of midface and orbital malformations on ocular outcomes in a large study population. A high pre-operative prevalence of ocular anomalies in patients with midface and/or orbital malformations was shown, which is in line with our previous work [3]. However, surgical correction of the midface had a direct and indirect substantial effect on several ocular outcomes, where both improvements and deteriorations were found, depending on the timing and type of surgery. Although various surgical corrections (OBO, LFIII, MB, FB) have been applied to correct midface and orbital malformations, little is known about their effect on ocular outcomes. Furthermore, timing is often delayed until older age due to diminished growth potential of the operated bone [20,21,22,23], but threatened function, either physically (vision problems, proptosis, OSAS, high ICP) or socially, can accelerate an operation.

### 4.1. Effect of Le Fort III on Ocular Outcomes

Literature is scarce on the effect of LFIII on ocular outcomes in patients with midfacial and orbital malformations. Minor ocular complications, such as ptosis (30%, *n* = 20) and strabismus (15%, *n* = 20), have been reported in the literature [12]. The latter is not in line with our study, where no effect was seen on strabismus after LFIII. In our study, VA improved in two patients with Apert syndrome and six patients with Crouzon syndrome. The main reasons for the improvement in VA were because the glasses were worn better (*n* = 2) as the position of the midface had improved, followed by the resolution of papilledema (*n* = 2), and an improvement of refractive errors (*n* = 1). No specific reasons were found for the improvement of VA in the other patients (*n* = 3). However, other reasons could be due to the improvement of proptosis, allowing the patients to close their eyelids (during sleep), leading to less dry eyes. Another possible explanation could be the young age of these patients at surgery (*n* = 1), making the measurement of VA less reliable, but more accurate when they get older. This is also described by Telleman et al., in which a high rate (32.1%, *n* = 8448) of failed and insufficient VA measurements at the age of 36 months was found [24]. Postoperatively, VA worsened in both eyes in one patient, which could be explained due to the known refraction amblyopia and opticopathy in both eyes. Finally, little effect was seen on postoperative binocular vision, only one patient had improvement of binocular vision, who was treated for refraction amblyopia, in whom also improvement of VA was seen.

### 4.2. Effect of Monobloc Advancement on Ocular Outcomes

The literature describes a 10% risk of corneal ulceration or keratitis after MB despite precautions [12,25], which is consistent with our study, where two patients (7.7%, *n* = 26) had corneal ulceration and keratitis due to insufficient closure of the eyelids during MB. Other possible reasons for postoperative corneal ulceration or keratitis may be due to postoperative edema or failure of satisfactory postoperative care [25]. Furthermore, the position of the eyes has been investigated in two studies after MB and both studies reported that the eyes moved forward [8,26,27]. It is remarkable that no change in vision has been identified by these studies, as stretching of the optic nerve may occur. In our study, VA improved in three patients with Apert syndrome and in three patients with Crouzon syndrome. In one case, VA improved as the glasses were consistently worn after surgery, followed by an improvement of lagophthalmus (*n* = 1), treatment for amblyopia (*n* = 2), and a decrease in hypermetropia (*n* = 1) and papilledema (*n* = 1). Furthermore, a general explanation for the improvement of VA after midface surgery can be related to a better position of the eyes in the orbits. In addition, VA worsened in four patients with Apert syndrome and in three patients with Crouzon syndrome. Possible explanations for the decrease in VA were worsened refraction (hypermetropia and astigmatism) leading to amblyopia (*n* = 1), followed by not wearing glasses after surgery as the frame did not fit (*n* = 4), and one patient was already known with a poor VA due to brain trauma. In a recent study, patients with syndromic craniosynostosis were treated by a combination of fronto-facial MB and FB, after which five patients (55.6%, *n* = 9) had an improvement of divergent strabismus (exotropia) without additional strabismus surgery [27]. These percentages were less in our study, where two patients (7.7%, *n* = 26) with Crouzon syndrome had an improvement of convergent (esotropia) and divergent (exotropia) strabismus after MB without additional strabismus surgery. A possible explanation could be that MB indirectly affects pre-existent strabismus by altering the position of the eyes or the eye muscles, or that the glasses are better worn; however, this has not been extensively studied in this study population.

### 4.3. Effect of Facial Bipartition on Ocular Outcomes

Facial bipartition has been indicated to correct hypertelorism when the functional correction of V-shaped malocclusion is required. Delaying surgery in severe hypertelorism may impair the development of binocular vision, adversely affecting long-term binocular vision after correction [28,29]. It has been stated that early surgery does not restore single binocular vision [30]. However, in our study, four patients (33%, *n* = 12) with CFNS had an improvement in their binocular vision after FB (age range 5.2–10.7 years). One of these patients had additional strabismus surgery after FB, therefore the improvement in binocular vision could be related to the strabismus and midface surgery. [31] Chen et al. also found an improvement in binocular vision in patients with craniofacial clefts and dysostosis after FB, which was, however, smaller than in our study, despite having almost the same age groups [31]. They hypothesized that this may be due to the age of surgery (6.8–7.7 years) and the influence of age on depth perception learning [31]. The effect of FB on strabismus has been described to varying degrees in the literature. In our study, six patients with CFNS (50%, *n* = 12) and one patient with Crouzon syndrome had worsened strabismus after FB. In only one patient with CFNS (8.3%, *n* = 12), the intermittent exotropia changed to accommodative esotropia, without any effect on their binocular vision. In general, a possible explanation for worsened strabismus may be a change in the position of the origo of the muscles (due to overmedialisation of the lateral orbital wall, which puts pressure on the belly of the lateral rectus). Our results are in line with the study of Greig et al., in which worsened postoperative strabismus has been reported in nine (45%, *n* = 20) patients with Apert and Crouzon syndromes who underwent FB [13]. These results are, however, in contrast to the study of Chen et al., where improvement of strabismus was seen after FB [31]. In the study of Chen et al., strabismus (exotropia) reduced from 83% to 29% in patients with craniofacial clefts (*n* = 34) and postoperatively reduced from 43% to 14% in patients with craniofacial dysostosis (*n* = 74) [31]. They reported that only 18% of the craniofacial clefts and 9% of the craniofacial dysostosis needed subsequent strabismus surgery [31].

### 4.4. Limitations and Future Suggestions

Studies describing a rare disease generally have limitations, which also applies to our study. The first limitation was the retrospective study design, which prevented us from evaluating all of the ophthalmic parameters we initially wanted to. In addition, not all patients had both pre-operative and postoperative ophthalmic measurements, or the follow-up period was too long so they could not be included. Moreover, the timing of postoperative ophthalmic measurements was not homogeneous for all patients, as the current guidelines do not contain specific moments to perform ophthalmic measurements after midface surgery. Therefore, the timing of postoperative measurements depended on whether there were complaints or if a patient was already being treated for a specific eye condition. Another limitation is the small relative sample sizes of the patients, as craniofacial disorders are rare. Despite the fact that we are one of the few studies that described the effect of midface surgery on ocular outcomes in such a large number of patients, we were still not able to draw firm conclusions. Finally, the ocular improvements or deteriorations were not always directly, but sometimes indirectly related to the effects of midface surgery, such as wearing glasses after surgery due to changes in the orbital skeleton, postoperative eye care, and young age at surgery, resulting in missing examinations. Future research should contain larger study samples using a multidisciplinary and multicenter approach, preferably internationally, in order to be able to draw firm conclusions for the different types of surgical techniques and disorders. This should be performed in a prospective manner, in which the ophthalmic conditions are pre- and postoperatively examined at least once by experienced ophthalmologists and orthoptists, to prevent any performance bias and loss in follow-up. Finally, future studies should focus on the causes of these ocular changes and should include 3D analyses of the orbital changes, hard-tissue, soft-tissue changes, and globe movements.

### 4.5. Clinical Suggestions

This study emphasizes the high prevalence of ocular anomalies in patients with syndromic craniosynostosis with both pre- and postoperative midface hypoplasia and/or orbital malformations, and therefore, we suggest proper ophthalmological and orthoptic examinations at least once pre- (0–6 months before midface surgery) and postoperatively (3–12 months after midface surgery), or earlier in case of (ocular) emergency. Furthermore, a worsening of strabismus was seen after MB and FB; therefore, we advise timely communication between the ophthalmologist and craniofacial surgeons with regard to strabismus surgery and midface surgery to avoid additional or multiple strabismus surgeries. Finally, both improvements and deteriorations were seen in VA after midface surgery, which could be partly explained due to correctly wearing glasses or noncompliance with occlusion therapy for amblyopia. Therefore, we advise discussing this matter with patients and their parents to create awareness. As some patients need occlusion therapy or glasses therapy, it is important that their treatment can be continued in a proper way after midface surgery, to prevent the development of amblyopia and decrease in VA.

## Figures and Tables

**Table 1:** Study characteristics.

Type of Surgery	Le Fort III (% or SD)	Monobloc (% or SD)	Facial Bipartition (% or SD)	Orbital Box (% or SD)	Total (% or SD)
**Number of patients**	20 (31.7)	26 (41.3)	15 (23.8)	2 (3.8)	63 (100.0)
**Sex (men:female)**	9 (45.0):11 (55.0)	14 (53.8):12 (46.2)	3 (20.0):12 (80.0)	0:2 (100.0)	26 (41.3):37 (58.7)
**Mean age surgery (y) ^1^**	11.8 (± 4.8)	7.8 (± 5.3)	7.9 (± 3.6)	17.4 (± 3.3)	9.4 (± 5.2)
**Orbital malformation**					
Hypertelorism	19 (95.0)	24 (92.3)	15 (100.0)	2 (100.0)	60 (95.2)
Midface hypoplasia	18 (90.0)	24 (92.3)	5 (33.3)	-	47 (74.6)
Vertical orbital dystopia	1 (5.0)	1 (3.8)	4 (26.7)	-	6 (9.5)
**Diagnosis**					
Apert	5 (25.0)	10 (38.5)	1 (6.7)	-	16 (25.4)
Crouzon	14 (70.0)	16 (61.5)	2 (13.3)	-	32 (50.8)
CFNS ^2^	1 (5.0)	-	12 (80.0)	2 (100.0)	15 (23.8)
**Operation indication**					
Proptosis	6 (30.0)	12 (46.2)	1 (6.7)	-	19 (30.2)
Elevated ICP ^3^	1 (5.0)	4 (15.4)	-	-	5 (5.9)
Malocclusion	19 (95.0)	21 (80.8)	3 (20.0)	-	43 (68.3)
OSAS ^4^	6 (30.0)	14 (53.8)	2 (13.3)	-	22 (34.9)
Esthetical	1 (5.0)	3 (11.5)	12 (80.0)	2 (100.0)	18 (28.6)

Abbreviations: ^1^ Y: years, ^2^ CFNS: craniofrontonasal dysplasia, ^3^ ICP: intracranial pressure, ^4^ OSAS: obstructive sleep apnea.

**Table 2:** Pre-operative ophthalmological examination.

	Apert (%)	Crouzon (%)	CFNS ^1^ (%)	Total (%)
**Number of patients**	16	32	15	63
**Strabismus type**				
Esotropia	4 (25.0)	4 (12.5)	3 (20.0)	11 (17.5)
Exotropia	6 (37.5)	13 (40.6)	8 (53.3)	27 (42.9)
Hypotropia	3 (18.8)	1 (3.1)	-	4 (6.3)
Hypertropia	-	1 (3.1)	1 (6.7)	2 (3.2)
V-pattern	16 (100)	32 (100)	14 (93.3)	62 (98.4)
**Visual acuity**				
Better eye (LogMAR)	0.25	0.10	0.25	0.16
Worse eye (LogMAR)	0.37	0.20	0.46	0.31
**Refractive errors**				
Anisometropia	7 (43.8)	8 (25.0)	8 (53.3)	23 (36.5)
Astigmatism	14 (87.5)	19 (59.4)	12 (80.0)	45 (71.4)
Hypermetropia	13 (81.3)	18 (56.3)	12 (80.0)	43 (68.3)
Myopia	2 (12.5)	4 (12.5)	-	6 (9.5)
**Binocular vision**				
Not present	4 (25.0)	1 (3.1)	4 (26.7)	9 (27.3)
Poor	2 (12.5)	2 (6.3)	4 (26.7)	8 (24.2)
Moderate	5 (31.3)	8 (25.0)	2 (13.3)	15 (45.5)
Good	-	1 (3.1)	-	1 (3.0)
**Amblyopia**	3 (18.8)	6 (18.8)	4 (26.7)	13 (20.6)
**Lacrimal system**				
Dryness	-	2 (6.3)	-	2 (3.2)
Tearing	3 (18.8)	5 (15.6)	-	8 (12.7)
NLDO ^2^	1 (6.3)	-	-	1 (1.6)
**Papilledema**				
Not present	13 (81.3)	25 (78.1)	15 (100)	53 (84.1)
Minimal	1 (6.3)	1 (3.1)	-	2 (3.2)
Low	2 (12.5)	2 (6.3)	-	4 (6.3)
Moderate	-	4 (12.5)	-	4 (6.3)

Abbreviations: ^1^ CFNS: craniofrontonasal dysplasia, ^2^ NLDO: nasolacrimal duct obstruction. Pm: note that the total prevalence of binocular vision, in the fourth column, was measured based on 33 patients.

**Table 3:** Ocular improvements after LFIII, MB, and FB.

Type of Surgery	Le Fort III					Monobloc					Facial Bipartition			
Type of Syndrome	Apert		Crouzon			Apert			Crouzon		Apert	Crouzon	CFNS	
**Age at surgery in years**	7–12	≥13	0–6	7–12	≥13	0–6	7–12	≥13	0–6	7–12	0–6	7–12	0–6	7–12
	*n* = 1	*n* = 3	*n* = 2	*n* = 8	*n* = 4	*n* = 3	*n* = 4	*n* = 3	*n* = 10	*n* = 5	*n* = 1	*n* = 2	*n* = 5	*n* = 6
**Strabismus type**														
Esotropia	-	-	-	-	-	-	-	-	1	-	-	-	-	-
Exotropia	-	-	-	-	-	-	-	-	1	-	-	-	-	-
Hyper-/hypotropia	-	-	-	-	-	-	-	-	-	-	-	-	-	-
**Visual acuity**														
1 LogMAR line	-	1	1	2	1	-	2	-	1	-	1	-	-	3
2 LogMAR lines	-	-	1	-	-	-	-	-	-	-	-	-	-	-
≥3 LogMAR lines	1	-	-	1	-	1	-	-	-	1	-	1	2	-
**Refractive errors**														
Astigmatism	-	-	-	-	-	-	-	-	1	-	-	-	-	-
Hypermetropia	-	-	1	1	-	1	-	-	1	-	-	-	-	-
Myopia	-	-	-	-	-	-	-	-	-	-	-	-	-	-
**Binocular vision**														
Not present to poor	-	-	-	-	-	-	-	-	-	-	-	-	1	-
Poor to moderate	-	-	-	-	-	-	-	-	-	1	-	-	-	3
Moderate to good	-	-	-	1	-	-	-	-	-	-	-	-	-	-
**Papilledema**														
Minimal to not present	-	-	-	1	-	1	-	-	-	-	-	-	-	-
Low to not present	-	-	1	1	-	1	-	1	-	-	-	-	-	-
Moderate to not present	-	-	-	-	-	-	-	-	1	1	-	-	-	-
Moderate to low	-	-	-	-	-	-	-	-	1	-	-	-	-	-

PM: note that the numbers in the table represent the number of patients with the ocular improvement.

**Table 4:** Ocular deteriorations after LFIII, MB, and FB.

Type of Surgery	Le Fort III			Monobloc					Facial Bipartition		
Type of syndrome	Apert		Crouzon	Apert			Crouzon		Crouzon	CFNS	
**Age at surgery in years**	7–12	≥13	7–12	0–6	7–12	≥13	0–6	7–12	7–12	0–6	7–12
	*n* = 1	*n* = 3	*n* = 8	*n* = 3	*n* = 4	*n* = 3	*n* = 10	*n* = 5	*n* = 2	*n* = 5	*n* = 6
**Strabismus type**											
Esotropia	-	-	-	-	-	-	1	-	1	-	4
Exotropia	-	-	-	-	-	-	-	-	-	-	1
Hyper-/hypotropia	-	-	-	-	-	-	-	-	-	2	-
**Visual acuity**											
1 LogMAR line	-	-	-	1	-	2	-	2	-	2	1
2 LogMAR lines	-	1	-	-	-	-	-	-	-	-	1
≥3 LogMAR lines	-	-	-	1	-	-	1	-	-	-	-
**Refractive errors**											
Astigmatism	1	-	-	1	1	-	3	-	-	-	2
Hypermetropia	-	-	-	1	1	-	1	-	-	-	-
Myopia	1	-	1	-	-	-	2	-	-	-	-
**Binocular vision**											
Poor to not present	-	-	-	-	-	-	-	-	-	-	-
Moderate to not present	-	-	-	-	-	-	-	-	-	-	1
Moderate to poor	-	-	-	1	-	-	-	-	-	-	-

PM: note that the numbers in the table represent the number of patients with the ocular deterioration.

## Data Availability

Data is described in Castor electronic database. Links to Castor electronic database could not be made public due to privacy restrictions, approved by the Institutional Review Board of Erasmus Medical Center, The Netherlands.

## Supplementary Materials

The following supporting information can be downloaded at: https://www.mdpi.com/article/10.3390/jcm12113862/s1.
