# Supplementary material for: Effect of Midface Surgery on Ocular Outcomes in Patients with Orbital and Midface Malformations
Source: J Clin Med. 2023 Jun 5;12(11):3862. doi: 10.3390/jcm12113862 (PMC10253425; doi:10.3390/jcm12113862)
Supplement: Supplementary file 1 [file jcm-12-03862-s001.zip › jcm-2384011-supplementary.pdf]

Supplementary Material A. An example of the orbital measurements

Anterior interorbital distance (AIOD)

Globe protrusion (GP) left and right eye

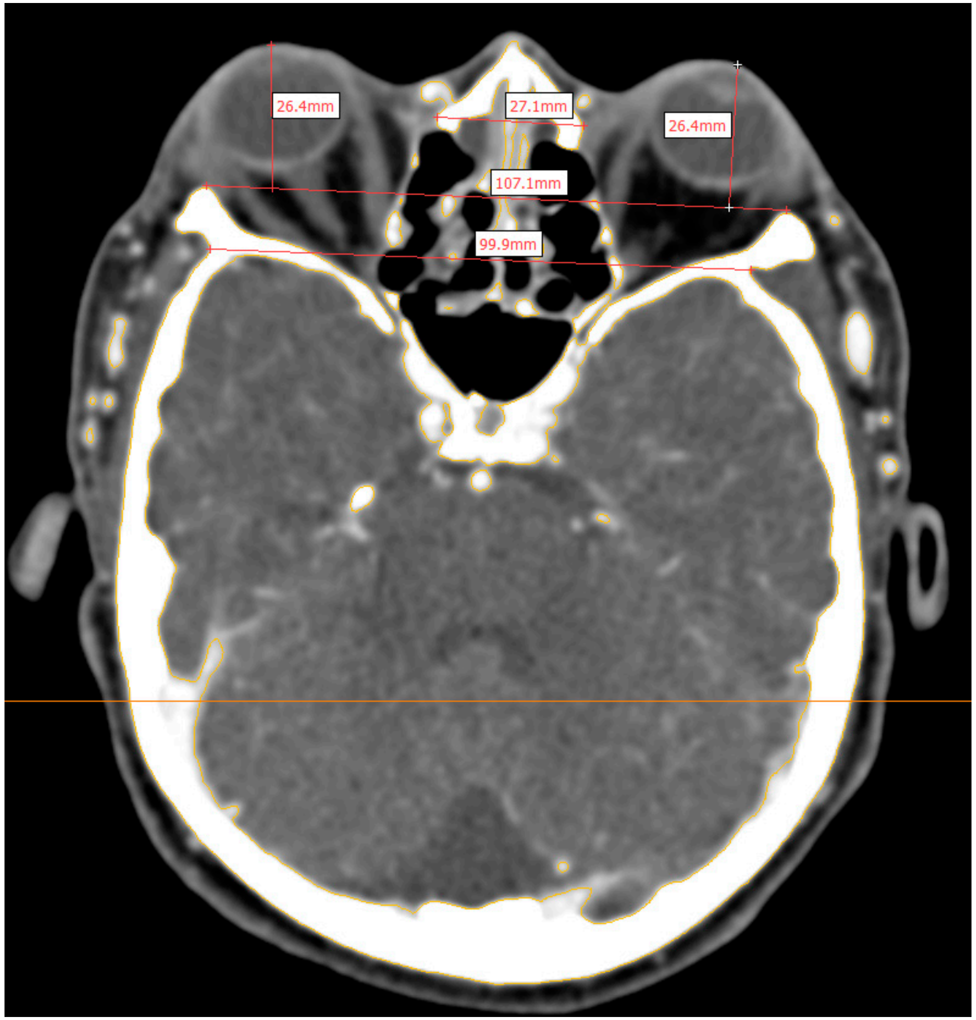

Supplementary Material B. Detailed patient characteristics and pre- and postoperative ophthalmological examinations sorted on age and disorder

Abbreviations:
OD: oculo dextra (right eye)
OS: oculo sinistra (left eye)
VA: visual acuity
NLDO: nasolacrimal duct obstruction
AIOD: anterior interorbital distance
GP OD: globe protrusion right eye
GP OS: globe protrusion left eye
RED: rigid external distraction
OSAS: obstructive sleep apnea syndrome
ICP: intracranial pressure
CFNS: craniofrontonasal dysplasia.

	Disorder Sex	Age surgery	Type surgery	Indication surgery	Pre-operative orbital measurements (mm)	Post-operative orbital measurements (mm)	Pre-operative ocular examination	Postoperative ocular examination	Effect surgery on ocular outcomes
1	Apert<br>Male	22.3	Monobloc + internal distraction	Malocclusion<br>OSAS<br>Esthetical	AIOD: 28.5<br>GP OD: 25.0<br>GP OS: 24.0	AIOD: 26.0<br>GP OD: 12.6<br>GP OS: 13.4	VA: OD 0.7, OS 0.7<br>Strabismus pattern: V<br>Strabismus type: not present<br>Refraction: hypermetropia, astigmatism<br>OD +4.00=C-3.25x100<br>OS +4.75=C-1.5x50<br>Binocular vision: not measured	VA: OD 0.8, OS 0.8<br>Strabismus pattern: V<br>Strabismus type: not present<br>Refraction: hypermetropia, astigmatism<br>OD +4.00=C-3.25x100<br>OS +4.75=C-1.5x50<br>Binocular vision: not measured	Improvements:<br>-<br>Deteriorations:<br>VA: 0.1 decrease ODS.<br><br>Explanation: Patient was known with a poor vision due to a car accident with brain trauma.
2	Apert<br>Female	19.3	Le Fort III + RED	Malocclusion	AIOD: 23.3<br>GP OD: 19.9<br>GP OS: 20.4	AIOD: 23.0<br>GP OD: 17.4<br>GP OS: 17.9	VA: OD 0.1, OS 0.05<br>Strabismus pattern: V<br>Strabismus type: not present<br>Refraction: astigmatism, hypermetropia<br>OD +1.50=C-1.75x96<br>OS +1.75=C-2.00x85<br>Binocular vision: poor	VA: OD 0.05, OS 0<br>Strabismus pattern: V<br>Strabismus type: not present<br>Refraction: astigmatism, hypermetropia<br>OD +1.50=C-1.75x85<br>OS +1.75=C-2.50x100<br>Binocular vision: poor	Improvements:<br>VA: ODS 0.05<br><br>Deteriorations:<br>-
3	Apert<br>Female	17.9	Monobloc + internal distraction	Malocclusion<br>Proptosis<br>Esthetical	AIOD: 25.6<br>GP OD: 20.5<br>GP OS: 20.8	AIOD: 20.3<br>GP OD: 10.1<br>GP OS: 12.6	VA: OD 0.7, OS 0.4<br>Strabismus pattern: V<br>Strabismus type: exotropia, hypotropia<br>Refraction: myopia, astigmatism<br>OD -1.5=C-3.25x30<br>OS -3.5=C-3.75x155<br>Binocular vision: not present	VA: OD 0.7, OS 0.5<br>Strabismus pattern: V<br>Strabismus type: exotropia, hypotropia<br>Refraction: myopia, astigmatism<br>OD -1.75=C-2.5x30<br>OS -4.0=C-4.0x155<br>Binocular vision: not present	Improvements:<br>-<br>Deteriorations:<br>VA: 0.1 decrease OS<br><br>Explanation: post-operative exposure keratitis OD. During monobloc OD remained dry. No reason for VA decrease OS could be found.

4	Apert<br>Female	16.8	Monobloc<br>+ internal<br>distraction	Malocclusion<br>Proptosis<br>Esthetical	AIOD: 27.1<br>GP OD: 26.4<br>GP OS: 26.4	AIOD: 26.3<br>GP OD: 19.0<br>GP OS: 18.7	VA: ODS 0<br>Strabismus pattern: V<br>Strabismus type: esotropia<br>Refraction: hypermetropia, astigmatism<br>OD +2.00=C-0.75x10<br>OS +2.00=C-1.00x152<br>Binocular vision: not measured<br>Neuro-ophthalmic: low papilledema	VA: ODS 0<br>Strabismus pattern: V<br>Strabismus type: esotropia<br>Refraction: hypermetropia, astigmatism<br>OD +2.00=C-0.75x10<br>OS +2.00=C-1.00x152<br>Binocular vision: not measured	Improvements:<br>No more papilledema<br><br>Deteriorations:<br>-
5	Apert<br>Male	15.9	Le Fort III<br>+ RED	OSAS<br>Malocclusion	AIOD: 28.1<br>GP OD: 17.7<br>GP OS: 19.4	AIOD: 28.0<br>GP OD: 14.8<br>GP OS: 17.4	VA: OD 0.3, OS 0.3<br>Strabismus pattern: V<br>Strabismus type: not present<br>Refraction: hypermetropia, astigmatism<br>OD +7.0=C-2.25x3<br>OS +7.50=C-1.75x15<br>Binocular vision: poor	VA: OD 0.4, OS 0.5<br>Strabismus pattern: V<br>Strabismus type: not present<br>Refraction: hypermetropia, astigmatism<br>OD +7.25=C-3.00x2<br>OS +7.25=C-2.75x10<br>Binocular vision: poor	Improvements:<br>-<br><br>Deteriorations:<br>VA OD decrease 0.1, OS decrease 0.2
6	Apert<br>Female	15	Le Fort III +<br>RED	Malocclusion<br>OSAS	AIOD: 30.8<br>GP OD: 18.7<br>GP OS: 22.1	AIOD: 30.1<br>GP OD: 17.6<br>GP OS: 19.3	VA: OD 0.15, OS 0.2<br>Strabismus pattern: V<br>Strabismus type: not present<br>Refraction: hypermetropia<br>OD +2.5=C-0.75x34<br>OS +4.5=C-0.5x38<br>Binocular vision: not measured	VA: OD 0.1, OS 0.1<br>Strabismus pattern: V<br>Strabismus type: not present<br>Refraction: hypermetropia, astigmatism<br>OD +2.50=C-1.50x22<br>OS + 5.0<br>Binocular vision: not measured	Improvements:<br>VA: Increase of 0.1 OS, increase 0.05 OD<br><br>Explanation: Did not wear glasses pre-operatively, but started to wear them postoperatively<br><br>Deteriorations:<br>-
7	Apert<br>Female	12	Le Fort III<br>+ RED	Malocclusion<br>Proptosis	AIOD: 25.5<br>GP OD: 20.5<br>GP OS: 19.5	AIOD: 24.8<br>GP OD: 17.8<br>GP OS: 16.4	VA: ODS 0.7<br>Strabismus pattern: V<br>Strabismus type: exotropia, hypotropia	VA: OD 0.5, OS 0.4<br>Strabismus pattern: V<br>Strabismus type: exotropia, hypotropia	Improvements:<br>VA 0.2 increase OD, 0.3 increase OS

							Refraction: myopia, astigmatism<br>OD -1.0=C-3.00x325<br>OS -3.00=c-3.50x160<br>Binocular vision: not present	Refraction: myopia, astigmatism<br>OD -2.00=C-4.00x25<br>OS -3.75=C-4.25x165<br>Binocular vision: not present	Explanation: VA improvement less proptosis post-op.<br><br>Deteriorations:<br>Increase myopia and astigmatism OD.
8	Apert<br>Male	10.3	Monobloc + internal distraction	Malocclusion	AIOD: 23.5<br>GP OD: 17.4<br>GP OS: 18.2	AIOD: 22.4<br>GP OD: 13.5<br>GP OS: 13.8	VA: OD 0.15, OS 0.5<br>Strabismus pattern: V<br>Strabismus type: exotropia, hypotropia<br>Refraction: hypermetropia, astigmatism<br>OD -0.50=C-1.00x99<br>OS +2.00=C-0.50x26<br>Binocular vision: moderate<br>Amblyopia: present OS<br>Torticollis: present	VA: OD 0.1, OS 0.4<br>Strabismus pattern: V<br>Strabismus type: exotropia, hypotropia<br>Refraction: hypermetropia, astigmatism<br>OD -0.50=C-1.50x90<br>OS +5.50=C-4.50x50<br>Binocular vision: moderate<br>Amblyopia: present OS<br>Torticollis: present	Improvements:<br>VA increase 0.05 OD, 0.1 OS<br><br>Explanation: VA OS improved due to amblyopia treatment<br><br>Deteriorations:<br>Increase of hypermetropia and astigmatism OS
9	Apert<br>Male	9.7	Monobloc + internal distraction	Malocclusion<br>Proptosis	AIOD: 27.8<br>GP OD: 21.3<br>GP OS: 19.5	AIOD: 23.7<br>GP OD: 12.1<br>GP OS: 11.4	VA: OD 0.3, OS 0.5<br>Strabismus pattern: V<br>Strabismus type: esotropia,<br>Refraction: astigmatism, hypermetropia<br>OD +4.00=C-2.75x20<br>OS +2.75=C-3.50x150<br>Binocular vision: not present<br>Eyelid: lagophthalmus	VA: OD 0.3, OS 0.4<br>Strabismus pattern: V<br>Strabismus type: esotropia,<br>Refraction: astigmatism, hypermetropia<br>OD +3.50=C-2.75x14<br>OS +2.50=C-3.00x166<br>Binocular vision: not present	Improvements:<br>VA OS 0.1 improved due to reduction of proptosis and lagophthalmus<br><br>Deteriorations:<br>Postoperative keratitis ODS due to air blown by oxygen mask during surgery.
10	Apert<br>Female	8.9	Monobloc + internal distraction	Malocclusion<br>OSAS	AIOD: 20.0<br>GP OD: 20.4<br>GP OS: 21.8	AIOD: 21.3<br>GP OD: 18.2<br>GP OS: 15.6	VA: ODS 0.4<br>Strabismus pattern: V<br>Strabismus type: exotropia	VA: ODS 0.4<br>Strabismus pattern: V<br>Strabismus type: exotropia	Improvements:<br>No more tearing of eyes due to correct closure eyelids.

							Refraction: hypermetropia, astigmatism<br>OD +3.00=C-2.50x125<br>OS +2.25=C-3.00x42<br>Binocular vision: moderate<br>Torticollis: present<br>Lacrimal: tearing eyes<br>Eyelid: lagophthalmus	Refraction: hypermetropia, astigmatism<br>OD +2.50=C-2.50x121<br>OS +3.00=C-3.00x25<br>Binocular vision: moderate<br>Torticollis: present	Lagophthalmus.<br><br>Deteriorations:<br>-
11	Apert<br>Female	8.3	Monobloc + internal distraction	Malocclusion<br>Proptosis	AIOD: 22.5<br>GP OD: 17.1<br>GP OS: 16.2	AIOD: 18.7<br>GP OD: 14.6<br>GP OS: 13.7	VA: OD 0, OS 0.1<br>Strabismus pattern: V<br>Strabismus type: exotropia,<br>Refraction: astigmatism<br>OD +0.25=C-0.50x130<br>OS +0.75=C-1.00x75<br>Binocular vision: not measured	VA: OD 0, OS 0.1<br>Strabismus pattern: V<br>Strabismus type: exotropia,<br>Refraction: astigmatism<br>OD +0.25=C-0.50x130<br>OS +0.75=C-1.00x75<br>Binocular vision: not measured	Improvements:<br>-<br><br>Deteriorations:<br>-
12	Apert<br>Male	6.2	Facial bipartition	Malocclusion	AIOD: 20.2<br>GP OD: 16.0<br>GP OS: 15.4	AIOD: 20.4<br>GP OD: 11.1<br>GP OS: 10.8	VA: OD 0.1, OS 0<br>Strabismus pattern: V<br>Strabismus type: not present<br>Refraction: hypermetropia<br>OD +3.75=C-0.5x110<br>OS +3.25=C-0.25x95<br>Binocular vision: moderate<br>Amblyopia: present OD	VA: ODS 0<br>Strabismus pattern: V<br>Strabismus type: not present<br>Refraction: hypermetropia<br>OD +3.00<br>OS +2.50=C-0.75x155<br>Binocular vision: moderate<br>Amblyopia: present OD	Improvements:<br>VA increase 0.1 OD<br><br>Explanation: VA improvement due to amblyopia treatment OD.<br><br>Deteriorations:<br>-

13	Apert<br>Male	5.4	Monobloc<br>+ internal<br>distraction	Malocclusion<br>OSAS<br>High ICP	AIOD: 15.8<br>GP OD: 19.1<br>GP OS: 18.9	AIOD: 17.5<br>GP OD: 15.6<br>GP OS: 15.2	VA: OD 0.5, OS 0.2<br>Strabismus pattern: V<br>Strabismus type: exotropia<br>Refraction: astigmatism, hypermetropia<br>OD +6.00=C-4.75 as 24<br>OS +5.00=C-3.50 as 170<br>Binocular vision: moderate<br>Amblyopia: present OD<br>Torticollis: present<br>Diplopia: present<br>Neuro-ophthalmic: Low papilledema<br>Lacrimal: tearing of eyes	VA: ODS 0.7<br>Strabismus pattern: V<br>Strabismus type: exotropia<br>Refraction: astigmatism, hypermetropia<br>OD +6.00=C-5.25x18<br>OS +5.25=C-5.25x170<br>Binocular vision: moderate<br>Amblyopia: present OD<br>Lacrimal: tearing of eyes	Improvements:<br>No more papilledema.<br>Torticollis.<br>Diplopia.<br><br>Deteriorations:<br>VA decrease 0.2 OD, 0.5 OS.<br>Astigmatism<br><br>Explanation: did not wear glasses postoperatively, increase of amblyopia and astigmatism reason for VA decrease.
14	Apert<br>Male	4.3	Monobloc<br>+ internal<br>distraction	Severe<br>proptosis	AIOD: 17.2<br>GP OD: 19.6<br>GP OS: 20.8	AIOD: 17.3<br>GP OD: 15.5<br>GP OS: 12.4	VA: OD 0.3, OS 0.1<br>Strabismus pattern: V<br>Strabismus type: not present<br>Refraction: astigmatism, hypermetropia<br>OD +2.75=C-2.00x30<br>OS +3.25=C-2.50x180<br>Binocular vision: moderate<br>Eyelid: lagophthalmus<br>Lacrimal: tearing of eyes	VA: OD 0.4, OS 0.2<br>Strabismus pattern: V<br>Strabismus type: not present<br>Refraction: astigmatism, hypermetropia<br>OD +3.75=C-2.00x20<br>OS +3.25=C-2.25x156<br>Binocular vision: poor<br>Lacrimal: tearing of eyes	Improvements:<br>Lagophthalmus<br><br>Deteriorations:<br>VA decrease 0.1 ODS.<br>Binocular vision<br>Hypermetropia OD.<br><br>Explanation: VA worsened due to amblyopia OD for which glasses therapy started.
15	Apert<br>Male	4	Monobloc<br>+ internal<br>distraction	Malocclusion<br>Proptosis<br>OSAS<br>High ICP	AIOD: 20.1<br>GP OD: 18.5<br>GP OS: 18.7	AIOD: 22.7<br>GP OD: 15.2<br>GP OS: 14.0	VA: OD 0.5, OS 0.3<br>Strabismus pattern: V<br>Strabismus type: esotropia<br>Refraction: hypermetropia, astigmatism	VA: ODS 0.1<br>Strabismus pattern: V<br>Strabismus type: esotropia<br>Refraction: hypermetropia, astigmatism	Improvements:<br>No more papilledema.<br>VA increase 0.4 OD, 0.2 OS.<br>Torticollis.

							OD +1.75=C-1.50x100<br>OS -0.25=C-3.25x160<br>Binocular vision: not present<br>Torticollis: present<br>Neuro-ophthalmic: minimal papilledema ODS	OD +0.50=C-1.75x30<br>OS 0.00=C-2.75x145<br>Binocular vision: not present	Hypermetropia OD.<br><br>Explanation: VA ODS improved due to improvement of papilledema and due to wearing new glasses postoperatively.<br><br>Deteriorations:<br>-
16	Apert<br>Male	7 months	Le Fort III + cranio-plasty	Proptosis<br>Malocclusion<br>Hydro-cephalus	AIOD: n.a.<br>GP OD: n.a.<br>GP OS: n.a.<br><br>No CT-scan available	AIOD: n.a.<br>GP OD: n.a.<br>GP OS: n.a.<br><br>No CT-scan available	VA: follows and fixes<br>Strabismus pattern: V<br>Strabismus type: esotropia<br>Refraction: hypermetropia, astigmatism<br>OD +2.50=C-1.25x90<br>OS +2.50=C-1.25x90<br>Binocular vision: not measured<br>Lacrimal: NLDO	VA: Follows and fixes<br>Strabismus pattern: V<br>Strabismus type: esotropia<br>Refraction: hypermetropia, astigmatism<br>OD +2.50=C-1.25x90<br>OS +2.50=C-1.25x90<br>Binocular vision: not measured<br>Lacrimal: NLDO	Improvements:<br>-<br><br>Deteriorations:<br>-
17	Crouzon<br>Male	18	Le Fort III + RED	Malocclusion	AIOD: 22.9<br>GP OD: 21.5<br>GP OS: 19.5	AIOD: 22.6<br>GP OD: 20.1<br>GP OS: 19.4	VA: ODS 0.1<br>Strabismus pattern: V<br>Strabismus type: esotropia<br>Refraction: hypermetropia, astigmatism<br>OD +3.75=C-3.50x89<br>OS +3.5=C-3.50x78<br>Binocular vision: not measured	VA: OD 0.0, OS 0.1<br>Strabismus pattern: V<br>Strabismus type: esotropia<br>Refraction: hypermetropia, astigmatism<br>OD +3.75=C-3.50X89<br>OS +3.5=C-3.50X78<br>Binocular vision: not measured	Improvements:<br>VA increase OD 0.1<br><br>Deteriorations:<br>-
18	Crouzon<br>Female	17.8	Le Fort III + RED	Malocclusion	AIOD: 23.5<br>GP OD: 21.1<br>GP OS: 22.3	AIOD: 20.6<br>GP OD: 12.9<br>GP OS: 14.7	VA: ODS -0.1<br>Strabismus pattern: V<br>Strabismus type: exotropia<br>Refraction: myopia, astigmatism	VA: OD ODS -0.1<br>Strabismus pattern: V<br>Strabismus type: exotropia<br>Refraction: myopia, astigmatism<br>OD -0.75=C-1.00x30<br>OS -1.75=C-1.25x140	Improvements:<br>-<br><br>Deteriorations:<br>Tearing of eyes

						OD -0.75=C-1.00x22<br>OS -1.75=C-1.25x138<br>Binocular vision: not measured	Lacrimal: tearing of eyes<br>Binocular vision: not measured	
19	Crouzon Female	15.7	Le Fort III + RED	Malocclusion	AIOD: 27.7<br>GP OD: 20.2<br>GP OS: 20.7	AIOD: 26.1<br>GP OD: 15.4<br>GP OS: 15.6	VA: ODS 0<br>Strabismus pattern: V<br>Strabismus type: not present<br>Refraction: ametropic<br>Binocular vision: not measured	VA: not measured<br>Strabismus pattern: V<br>Strabismus type: present<br>Refraction: ametropic<br>Binocular vision: not measured	Improvements:<br>-<br><br>Deteriorations:<br>-
20	Crouzon Male	15.7	Monobloc + internal distraction	High ICP OSAS	AIOD: n.a.<br>GP OD: n.a.<br>GP OS: n.a.<br><br>No CT-scan available	AIOD: n.a.<br>GP OD: n.a.<br>GP OS: n.a.<br><br>No CT-scan available	VA: ODS 0<br>Strabismus pattern: V<br>Strabismus type: not present<br>Refraction:<br>OD +0.25=C-0.50x1<br>OS +0.25=C-0.50x145<br>Binocular vision: moderate	VA: ODS 0<br>Strabismus pattern: V<br>Strabismus type: not present<br>Refraction:<br>OD +0.25=C-0.50x148<br>OS+ 0.25=C-0.50x160<br>Binocular vision: moderate	Improvements:<br>-<br>Deteriorations:<br>3 days postoperatively subconjunctival bleeding with chemosis OD, for which therapy started
21	Crouzon Female	14.2	Le Fort III + RED	Malocclusion Proptosis	AIOD: 23.6<br>GP OD: 19.0<br>GP OS: 19.9	AIOD: 23.9<br>GP OD: 16.5<br>GP OS: 17.5	VA: ODS 0<br>Strabismus pattern: V<br>Strabismus type: not present<br>Refraction: hypermetropia, astigmatism<br>OD +5.0= C-3.50x75<br>OS +4.0=C-0.75x90<br>Binocular vision: moderate	VA: ODS 0<br>Strabismus pattern: V<br>Strabismus type: not present<br>Refraction: hypermetropia, astigmatism<br>OD +5.25=C-3.50x75<br>OS +3.75=C-0.25x125<br>Binocular vision: moderate	Improvements:<br>-<br><br>Deteriorations:<br>-
22	Crouzon Male	12.3	Le Fort III + RED and internal distraction	Elevated ICP OSAS	AIOD: 26.2<br>GP OD: 23.0<br>GP OS: 17.4	AIOD: 22.3<br>GP OD: 18.8<br>GP OS: 14.3	VA: OD 0.15, OS 0.7<br>Strabismus pattern: V<br>Strabismus type: exotropia<br>Refraction: astigmatism<br>OD -0.25=C-1.00x32<br>OS -0.25=C-1.25x58<br>Binocular vision: not measured<br>Neuro-ophthalmic: moderate papilledema	VA: OD 0.05 OS 0.7<br>Strabismus pattern: V<br>Strabismus type: exotropia<br>Refraction: astigmatism<br>OD -0.25=C-1.00x32<br>OS -0.25=C-1.25x58<br>Binocular vision: not measured	Improvements:<br>VA increase of 0.1 OD due to decrease papilledema.<br><br>Deteriorations:<br>-

								Neuro-ophthalmic: moderate papilledema (remained stable)	
23	Crouzon<br>Male	11.8	Le Fort III<br>+ RED	Malocclusion<br>OSAS	AIOD: n.a.<br>GP OD: n.a.<br>GP OS: n.a.<br><br>No CT-scan available	AIOD: n.a.<br>GP OD: n.a.<br>GP OS: n.a.<br><br>No CT-scan available	VA: OD 0.05, OS 0.5<br>Strabismus pattern: V<br>Strabismus type: not present<br>Refraction: myopia, astigmatism<br>OD -1.00=C-1.25x135<br>OS 0.00<br>Binocular vision: not measured	VA: not measured<br>Strabismus pattern: V<br>Strabismus type: not present<br>Refraction: myopia, astigmatism<br>OD -1.00=C-1.25x135<br>OS 0.00<br>Binocular vision: not measured	Improvements:<br>-<br><br>Deteriorations:<br>-
24	Crouzon<br>Female	11.7	Le Fort III<br>+ RED	Malocclusion	AIOD: n.a.<br>GP OD: n.a.<br>GP OS: n.a.<br><br>No CT-scan available	AIOD: n.a.<br>GP OD: n.a.<br>GP OS: n.a.<br><br>No CT-scan available	VA: OD 0.3, OS 1.0<br>Strabismus pattern: V<br>Strabismus type: exotropia<br>Refraction: myopia, astigmatism<br>Binocular vision: not measured<br>Lacrimal: tearing of eyes<br>Amblyopia: present OS	VA: OD 0.3, OS 1.0<br>Strabismus pattern: V<br>Strabismus type: exotropia<br>Refraction: myopia, astigmatism<br>Binocular vision: not measured<br>Amblyopia: present OS	Improvements:<br>Tearing of eyes<br><br>Deteriorations:<br>-
25	Crouzon<br>Male	10.3	Monobloc without distraction	Malocclusion<br>Proptosis	AIOD: 24.7<br>GP OD: 20.3<br>GP OS: 21.9	AIOD: 25.6<br>GP OD: 16.6<br>GP OS: 17.3	VA: ODS: 0<br>Strabismus pattern: V<br>Strabismus type: exotropia<br>Refraction: hypermetropia, astigmatism<br>OD +0.75=C-0.50x6<br>OS +1.50=C-1.75x28<br>Binocular vision: moderate<br>Torticollis: present	VA: OD 0.15, OS 0<br>Strabismus pattern: V<br>Strabismus type: exotropia<br>Refraction: astigmatism<br>OD 0.00=C-1.25x180<br>OS +0.75=C-1.00x22<br>Binocular vision: moderate<br>Torticollis: present	Improvements:<br>-<br>Deteriorations:<br>VA decrease of 0.15 OD.<br>Diplopia for one month.<br><br>Explanation: eye dryness complaints and not wearing glasses postoperatively.
26	Crouzon<br>Male	10.2	Le Fort III<br>+ RED	Malocclusion<br>Proptosis	AIOD: 30.4<br>GP OD: 21.4<br>GP OS: 20.2	AIOD: 28.4<br>GP OD: 15.8<br>GP OS: 13.9	VA: ODS 0<br>Strabismus pattern: V<br>Strabismus type: exotropia<br>Refraction: ODS +0.50	VA: ODS 0<br>Strabismus pattern: V<br>Strabismus type: exotropia<br>Refraction: ODS +0.50	Improvements:<br>-<br><br>Deteriorations:

							Binocular vision: not measured	Binocular vision: not measured<br>Diplopia: present for one month	Diplopia for one month
27	Crouzon<br>Female	9.6	Monobloc<br>+ internal distraction	Malocclusion<br>Proptosis<br>OSAS	AIOD: 20.7<br>GP OD: 17.6<br>GP OS: 21.3	AIOD: 20.8<br>GP OD: 9.8<br>GP OS: 11.0	VA: OD 0.1, OS 0.5<br>Strabismus pattern: V<br>Strabismus type: exotropia<br>Refraction: hypermetropia, astigmatism<br>OD +4.5=C-2.50x19<br>OS +3.50=C-2.00x163<br>Binocular vision: not present<br>Amblyopia: present OS	VA: OD 0.1, OS 0.1<br>Strabismus pattern: V<br>Strabismus type: exotropia<br>Refraction: hypermetropia, astigmatism<br>OD +3.75=C-2.00x28<br>OS +3.75=C-3.50x150<br>Binocular vision: not present<br>Amblyopia: improved OS	Improvements:<br>VA increase 0.4 OS<br><br>Explanation: VA improved due to occlusion therapy.<br><br>Deteriorations:<br>-
28	Crouzon<br>Female	9.6	Le Fort III<br>+ RED	Malocclusion<br>Proptosis	AIOD: 21.9<br>GP OD: 18.6<br>GP OS: 20.1	AIOD: 20.6<br>GP OD: 12.3<br>GP OS: 14.5	VA: ODS 0.5<br>Strabismus pattern: V<br>Strabismus type: not present<br>Refraction: myopia, astigmatism<br>OD -7=C-3.00x20<br>OS -7=C-4.00x170<br>Binocular vision: moderate	VA: ODS 0.15<br>Strabismus pattern: V<br>Strabismus type: not present<br>Refraction: myopia, astigmatism<br>OD -9.5=C-3.0x110<br>OS -8.75=C-4.00x162<br>Binocular vision: good	Improvements:<br>VA increase 0.35 ODS.<br>Binocular vision from moderate to good.<br><br>Deteriorations:<br>Myopia ODS
29	Crouzon<br>Male	8.9	Le Fort III<br>+ RED	Malocclusion<br>OSAS	AIOD: 25.2<br>GP OD: 19.8<br>GP OS: 18.4	AIOD: 26.1<br>GP OD: 12.8<br>GP OS: 9.1	VA: ODS 0<br>Strabismus pattern: V<br>Strabismus type: not present<br>Refraction: ametropic<br>Binocular vision: not measured<br>Neuro-ophthalmic: Low papilledema	VA: ODS 0<br>Strabismus pattern: V<br>Strabismus type: not present<br>Refraction: ametropic<br>Binocular vision: not measured	Improvements:<br>No more papilledema<br><br>Deteriorations:<br>-
30	Crouzon<br>Female	8.6	Facial bipartition<br>+ external distraction	Malocclusion<br>Proptosis<br>OSAS	AIOD: 27.5<br>GP OD: 18.8<br>GP OS: 20.7	AIOD: 17.0<br>GP OD: 12.9<br>GP OS: 14.8	VA: OD 0.3, OS 0.3<br>Strabismus pattern: V<br>Strabismus type: exophoria OD<br>Refraction: hypermetropia<br>OD +4.25=C-0.75x67<br>OS +4.0=C-0.5x13<br>Binocular vision: moderate	VA: ODS 0<br>Strabismus pattern: V<br>Strabismus type: esotropia OD<br>Refraction: hypermetropia<br>OD +4.00=C-0.75x28<br>OS +3.25<br>Binocular vision: moderate	Improvement<br>VA increase of 0.3 ODS.<br><br>Deteriorations:<br>Torticollis.<br>Post-op developed esotropia OD, pre-

								torticollis: present	op this was exophoria.<br>However no effect on binocular vision.
31	Crouzon<br>Female	8.4	Monobloc<br>+ internal distraction	Malocclusion	AIOD: 21.4<br>GP OD: 18.2<br>GP OS: 19.1	AIOD: 16.7<br>GP OD: 15.4<br>GP OS: 16.4	VA: OD 0.05, OS -0.1<br>Strabismus type: not present<br>Refraction: hypermetropia<br>OD +1.5<br>OS +1.5<br>Binocular vision: not measured	VA: OD 0.05, OS -0.1<br>Strabismus type: not present<br>Refraction: hypermetropia<br>OD +1.5<br>OS +1.5<br>Binocular vision: not measured	Improvements:<br>-<br><br>Deteriorations:<br>-
32	Crouzon<br>Male	8.2	Le Fort III<br>+ RED	Malocclusion	AIOD: 25.3<br>GP OD: 22.8<br>GP OS: 21.5	AIOD: 26.9<br>GP OD: 22.2<br>GP OS: 20.0	VA: OD 0.05, OS 0.1<br>Strabismus pattern: V<br>Strabismus type: not present<br>Refraction: hypermetropia<br>OD +1.75=C-0.25x135<br>OS +2.00=C-0.50x25<br>Binocular vision: good	VA: ODS: 0<br>Strabismus pattern: V<br>Strabismus type: not present<br>Refraction:<br>OD +0.50<br>OS +0.50=C-0.50x9<br>Binocular vision: good	Improvements:<br>VA increase OD 0.05, OS 0.1.<br>Hypermetropia ODS.<br><br>Explanation: started wearing glasses postoperatively.<br><br>Deteriorations:<br>-
33	Crouzon<br>Female	8	Le Fort III<br>+ RED	Malocclusion<br>Proptosis	AIOD: 20.0<br>GP OD: 18.4<br>GP OS: 20.8	AIOD: 20.9<br>GP OD: 17.0<br>GP OS: 15.2	VA: OD 0, OS 0.05<br>Strabismus pattern: V<br>Strabismus type: esotropia<br>Refraction: hypermetropia<br>OD +3.0=C-0.50x105<br>OS +3.0<br>Binocular vision: not measured<br>Neuro-ophthalmic: minimal papilledema<br>Lacrimal: tearing of eyes	VA: OD 0, OS 0.05<br>Strabismus pattern: V<br>Strabismus type: esotropia<br>Refraction: hypermetropia<br>OD +3.0=C-0.50x105<br>OS +3.0<br>Binocular vision: not measured<br>Lacrimal: tearing of eyes	Improvements:<br>No more papilledema.<br>Less tearing of eyes, still present.<br><br>Deteriorations:<br>-
34	Crouzon<br>Male	7.9	Monobloc<br>+ internal distraction	Malocclusion<br>OSAS	AIOD: 20.7<br>GP OD: 20.3<br>GP OS: 19.3	AIOD: 20.9<br>GP OD: 14.5<br>GP OS: 14.5	VA: ODS 0.2<br>Strabismus pattern: V<br>Strabismus type: exotropia<br>Refraction: astigmatism	VA: ODS 0.15<br>Strabismus pattern: V<br>Strabismus type: exotropia<br>Refraction: astigmatism	Improvements:<br>VA increase of 0.05 ODS.

						OD +0.75=C-1.00x70<br>OS 0.00=C-1.00x95<br>Binocular vision: moderate<br>Amblyopia: present	OD +0.50=C-1.00x85<br>OS -0.25=C-0.75x65<br>Binocular vision: moderate<br>Amblyopia: present	Deteriorations:<br>-
35	Crouzon<br>Female	7	Monobloc<br>+ internal<br>distraction	Malocclusion<br>OSAS<br>High ICP	AIOD: 23.1<br>GP OD: 22.3<br>GP OS: 23.1	AIOD: 23.3<br>GP OD: 15.6<br>GP OS: 17.2	VA: OD 0,1 OS 0.15<br>Strabismus pattern: V<br>Strabismus type: esotropia, hypotropia<br>Refraction: hypermetropia, astigmatism<br>OD +0.25=C-2.75x65<br>OS +1.25=C-3.5x135<br>Binocular vision: poor<br>Neuro-ophthalmic: moderate papilledema	VA: ODS 0.2<br>Strabismus pattern: V<br>Strabismus type: esotropia, hypotropia<br>Refraction: hypermetropia, astigmatism<br>OD -1.0=C-2.0x62<br>OS +1.00=C-3.50X150<br>Binocular vision: moderate	Improvements:<br>Binocular vision from poor to moderate.<br>No more papilledema.<br><br>Deteriorations:<br>VA decrease 0.1 OD, 0.05 OS.
36	Crouzon<br>Female	6.9	Monobloc<br>+ internal<br>distraction	Proptosis<br>OSAS	AIOD: n.a.<br>GP OD: n.a.<br>GP OS: n.a.<br><br>No CT-scan available	AIOD: n.a.<br>GP OD: n.a.<br>GP OS: n.a.<br><br>No CT-scan available	VA: ODS 0.1<br>Strabismus pattern: V<br>Strabismus type: not present<br>Refraction: hypermetropia, astigmatism<br>OD +1.25=C-0.75x10<br>OS +1.00=C-1.00x175<br>Binocular vision: moderate	VA: ODS 0<br>Strabismus pattern: V<br>Strabismus type: not present<br>Refraction: hypermetropia<br>OD +1.00=C-0.75x10<br>OS +1.00=C-0.75x165<br>Binocular vision: moderate	Improvements:<br>VA increase of 0.1 ODS.<br><br>Deteriorations:<br>-
37	Crouzon<br>Male	5.6	Le Fort III<br>+ RED and internal distraction	Severe OSAS<br>Malocclusion	AIOD: 25.4<br>GP OD: 20.9<br>GP OS: 20.7	AIOD: 25.1<br>GP OD: 18.7<br>GP OS: 18.7	VA: ODS 0.1<br>Strabismus pattern: V<br>Strabismus type: not present<br>Refraction: ametropic<br>Binocular vision: moderate	VA: ODS 0<br>Strabismus pattern: V<br>Strabismus type: not present<br>Refraction: ametropic<br>Binocular vision: not measured	Improvements:<br>VA increase 0.1 ODS.<br><br>Deteriorations:<br>-
38	Crouzon<br>Female	5	Le Fort III<br>+ RED	Malocclusion	AIOD: n.a.<br>GP OD: n.a.<br>GP OS: n.a.<br><br>No CT-scan available	AIOD: n.a.<br>GP OD: n.a.<br>GP OS: n.a.<br><br>No CT-scan available	VA: ODS 0.3<br>Strabismus pattern: V<br>Strabismus type: not present<br>Refraction: hypermetropia, astigmatism<br>OD +1.5=C-3.5x25<br>OS +1.5=C-4.0x50<br>Binocular vision: not measured	VA: ODS 0.1<br>Strabismus pattern: V<br>Strabismus type: not present<br>Refraction: astigmatism<br>OD +0.5=C-4.25x15<br>OS +0.5=C-4.25x170<br>Binocular vision: not measured<br>Diplopia: present	Improvements:<br>No more papilledema.<br>VA increase 0.2 ODS.<br>Hypermetropia improved ODS.<br><br>Deteriorations:

							Neuro-ophthalmic: low papilledema		Diplopia for one month postoperatively.
39	Crouzon<br>Male	4.3	Monobloc<br>+ internal distraction	Malocclusion<br>Proptosis	AIOD: 21.3<br>GP OD: 20.1<br>GP OS: 19.8	AIOD: 17.4<br>GP OD: 12.4<br>GP OS: 13.1	VA: not measured<br>Strabismus pattern: V<br>Strabismus type: exotropia<br>Refraction: hypermetropia<br>OD +0.75<br>OS +1.00<br>Lacrimal: tearing of eyes<br>Binocular vision: not measured	VA: OD 0.4, OS 0.15<br>Strabismus pattern: V<br>Strabismus type: exotropia<br>Refraction: hypermetropia, astigmatism<br>OD +1.75=C-2.75x34<br>OS +2.25=C=2.25x152<br>Binocular vision: not measured	Improvements:<br>Tearing of eyes.<br>Exotropia OS reduced postoperatively, no more manifest strabismus.<br><br>Deteriorations:<br>Hypermetropia and astigmatism ODS
40	Crouzon<br>Female	4	Monobloc<br>Without distraction	Malocclusion	AIOD: n.a.<br>GP OD: n.a.<br>GP OS: n.a.<br><br>No CT-scan available	AIOD: n.a.<br>GP OD: n.a.<br>GP OS: n.a.<br><br>No CT-scan available	VA: OD 0.3, OS 0.2<br>Strabismus pattern: V<br>Strabismus type: esotropia<br>Refraction: hypermetropia, astigmatism<br>OD +2.25=C-4.00x173<br>OS +2.50=C-3.75x176<br>Binocular vision: not measured<br>Amblyopia: present OD<br>Torticollis: present	VA: OD 0.3, OS 0.6<br>Strabismus pattern: V<br>Strabismus type: esotropia<br>Refraction: hypermetropia, astigmatism<br>OD -1.00=C-0.50x141<br>OS +2.50=C-4.75x180<br>Binocular vision: not measured<br>Amblyopia: present ODS<br>Torticollis: present	Improvements:<br>Refraction change OD.<br>Astigmatism OD.<br>Esotropia OS reduced, still present.<br>Refraction amblyopia improved OD.<br><br>Deteriorations:<br>Esotropia OD, was not present pre-operatively.<br>VA decrease 0.4 OS.<br>Amblyopia OS increased, does not wear glasses.
41	Crouzon<br>Male	4	Monobloc<br>+ internal distraction	Eyeball luxation due to proptosis<br>OSAS	AIOD: 23.5<br>GP OD: 20.9<br>GP OS: 20.9	AIOD: 20.2<br>GP OD: 17.1<br>GP OS: 17.5	VA: ODS 0<br>Strabismus pattern: V<br>Strabismus type: exotropia<br>Refraction: hypermetropia, astigmatism	VA: OD 0, OS 0.0<br>Strabismus pattern: V<br>Strabismus type: exotropia<br>Refraction: hypermetropia, astigmatism	Improvements:<br>-<br><br>Deteriorations:<br>-

							OD +1.25=C-1.00x100<br>OS +1.25=C-2.50x75<br>Binocular vision: not measured	OD +0.25=C-0.50x127<br>OS +1.00=C-1.75x84<br>Binocular vision: not measured	
42	Crouzon<br>Male	3.5	Monobloc<br>+ internal<br>distraction	Malocclusion<br>OSAS	AIOD: 14.8<br>GP OD: 21.5<br>GP OS: 21.2	AIOD: 15.3<br>GP OD: 12.3<br>GP OS: 11.3	VA: not measured<br>Strabismus pattern: V<br>Strabismus type: exotropia<br>Refraction: hypermetropia<br>OD +0.75<br>OS +1.25=C-0.50x110<br>Binocular vision: not measured<br>Lacrimal: tearing of eyes	VA: not measured<br>Strabismus pattern: V<br>Strabismus type: exotropia<br>Refraction: hypermetropia,<br>OD +0.75<br>OS +1.25=C-0.50x110<br>Binocular vision: not measured<br>Lacrimal: tearing of eyes	Improvements:<br>-<br><br>Deteriorations:<br>-
43	Crouzon<br>Male	3.3	Monobloc<br>+ internal<br>distraction	OSAS	AIOD: 27.8<br>GP OD: 17.8<br>GP OS: 19.4	AIOD: 29.4<br>GP OD: 16.8<br>GP OS: 17.0	VA: not measured<br>Strabismus pattern: V<br>Strabismus type: exotropia<br>Refraction: hypermetropia, astigmatism<br>OD +4.50=C-1.75x15<br>OS +2.00=C-1.50x180<br>Binocular vision: not measured<br>Lacrimal: dryness complaints	VA: not measured<br>Strabismus pattern: V<br>Strabismus type: exotropia<br>Refraction: hypermetropia, astigmatism<br>OD +4.50=C-1.75x13<br>OS +2.00=C-1.50x175<br>Binocular vision: not measured	Improvements:<br>Dryness complaints.<br><br>Deteriorations:<br>-
44	Crouzon<br>Male	3.3	Monobloc<br>+ internal<br>distraction	Malocclusion	AIOD: n.a.<br>GP OD: n.a.<br>GP OS: n.a.<br><br>No CT-scan available	AIOD: n.a.<br>GP OD: n.a.<br>GP OS: n.a.<br><br>No CT-scan available	VA: not measured<br>Strabismus pattern: V<br>Strabismus type: not present<br>Refraction: astigmatism<br>OD -0.50=C-0.50x60<br>OS 0.00=C-3.50x140<br>Amblyopia: present<br>Binocular vision: not measured	VA: not measured<br>Strabismus pattern: V<br>Strabismus type: not present<br>Refraction: myopia, astigmatism<br>OD -2.00=C-2.00x45<br>OS -1.25=C-4.00x125<br>Amblyopia: present<br>Binocular vision: not measured	Improvements:<br>-<br><br>Deteriorations:<br>Myopia ODS.<br>Astigmatism OD.
45	Crouzon<br>Female	2.8	Monobloc<br>+ internal<br>distraction	Malocclusion<br>High ICP	AIOD: 22.3<br>GP OD: 21.5<br>GP OS: 20.9	AIOD: 19.4<br>GP OD: 11.0<br>GP OS: 14.2	VA: not measured<br>Strabismus pattern: V<br>Strabismus type: exotropia<br>Refraction:	VA: ODS 0.1<br>Strabismus pattern: V<br>Strabismus type: exotropia	Improvements:<br>Papilledema<br><br>Deteriorations:

							OD -0.25=C-0.75x180<br>OS +0.75<br>Binocular vision: not measured<br>Amblyopia: present<br>Torticollis: present<br>Neuro-ophthalmic: moderate papilledema	Refraction: hypermetropia, astigmatism<br>OD -0.50=C-2.50x30<br>OS +1.50=C-1.50x175<br>Binocular vision: not measured<br>Amblyopia: present<br>Torticollis: present<br>Neuro-ophthalmic: low papilledema	Astigmatism ODS.<br>Enophthalmus.
46	Crouzon<br>Male	2.3	Monobloc + internal distraction	Malocclusion<br>OSAS	AIOD: 23.1<br>GP OD: 24.7<br>GP OS: 24.2	AIOD: 21.4<br>GP OD: 17.5<br>GP OS: 20.4	VA: Fixes and follows<br>Strabismus pattern: V<br>Strabismus type: exotropia, hypertropia<br>Refraction: hypermetropia, astigmatism<br>OD +1.50=C-2.00x90<br>OS +1.50=C-1.50x45<br>Binocular vision: not measured<br>Lacrimal: tearing of eyes<br>Neuro-ophthalmic: moderate papilledema	VA: Fixes and follows<br>Strabismus pattern: V<br>Strabismus type: exotropia, hypertropia<br>Refraction: hypermetropia, astigmatism<br>OD +1.50=C-1.50x180<br>OS +1.50=C-1.00x180<br>Binocular vision: not measured<br>Lacrimal: tearing of eyes	Improvements:<br>No more papilledema.<br><br>Deteriorations:<br>-
47	Crouzon<br>Female	2.1	Facial bipartition	Malocclusion<br>OSAS	AIOD: 17.0<br>GP OD: 22.4<br>GP OS: 17.7	AIOD: 14.5<br>GP OD: 10.1<br>GP OS: 12.3	VA: not able to measure<br>Strabismus pattern: V<br>Strabismus type: not present<br>Refraction: hypermetropia, astigmatism<br>OD +5.00=C-2.75x34<br>OS +4.75=C-2.75x140<br>Binocular vision: poor<br>Eyelid: lagophthalmus	VA: not able to measure<br>Strabismus pattern: V<br>Strabismus type: not present<br>Refraction: hypermetropia, astigmatism<br>OD +5.00=C-2.75x34<br>OS +5.00=C-2.75x149<br>Binocular vision: poor	Improvements:<br>Lagophthalmus<br><br>Deteriorations:<br>-
48	Crouzon<br>Female	1	Monobloc + internal distraction	Proptosis<br>Malocclusion<br>OSAS	AIOD: 12.8<br>GP OD: 20.0<br>GP OS: 19.5	AIOD: 13.4<br>GP OD: 17.8<br>GP OS: 18.3	VA: fix and follow good<br>Strabismus type: not present<br>Refraction: ametropic<br>Binocular vision: not measured	VA: OD fix and follow good<br>Strabismus type: not present<br>Refraction: ametropic<br>Binocular vision: not measured	Improvements:<br>Lagophthalmus.<br>Dryness complaints.<br><br>Deteriorations:<br>-

							Lacrimal: dryness complaints		
49	CFNS<br>Female	20.3	Orbital box osteotomy	Esthetical	AIOD: 30.0<br>GP OD: 15.8<br>GP OS: 15.6	AIOD: 23.8<br>GP OD: 15.8<br>GP OS: 15.3	VA: OD 0.5, OS 0.7<br>Strabismus pattern: V<br>Strabismus type: not present<br>Refraction: hypermetropia, astigmatism<br>OD +5.25=C-4.25x120<br>OS+5.5=C-8.00x50<br>Binocular vision: not present<br>Amblyopia: present	VA: OD 0.5, OS 0.7<br>Strabismus pattern: V<br>Strabismus type: not present<br>Refraction: hypermetropia, astigmatism<br>OD +5.00=C-4.50x127<br>OS +5.25=C-8.25x48<br>Binocular vision: not present<br>Amblyopia: present	Improvements:<br>-<br><br>Deteriorations:<br>-
50	CFNS<br>Male	17.5	Facial bipartition	Esthetical	AIOD: 30.6<br>GP OD: 18.1<br>GP OS: 18.4	AIOD: 27.2<br>GP OD:16.8<br>GP OS: 16.8	VA: ODS 0.05<br>Strabismus type: not present<br>Refraction: ametropic<br>Binocular vision: not measured	VA: ODS 0.05<br>Strabismus type: not present<br>Refraction: ametropic<br>Binocular vision: not measured	Improvements:<br>-<br><br>Deteriorations:<br>-
51	CFNS<br>Female	16.7	Le Fort III without distraction	Esthetical<br>Malocclusion	AIOD: 21.1<br>GP OD: 14.4<br>GP OS: 16.1	AIOD: 18.6<br>GP OD: 8.6<br>GP OS: 11.2	VA: OD 1.5, OS 0.6<br>Strabismus pattern: V<br>Strabismus type: not present<br>Refraction: ametropic<br>Amblyopia: present OD<br>Torticollis: present<br>Binocular vision: not measured	VA: OD 1.5, OS 0.6<br>Strabismus pattern: V<br>Strabismus type: not present<br>Refraction: ametropic<br>Amblyopia: present OD<br>Torticollis: present<br>Lacrimal: dryness complaints<br>Binocular vision: not measured	Improvements:<br>-<br>Deteriorations:<br>Dryness complaints.
52	CFNS<br>Female	14.5	Orbital box osteotomy	Esthetical	AIOD: n.a.<br>GP OD: n.a.<br>GP OS: n.a.<br><br>No CT-scan available	AIOD: n.a.<br>GP OD: n.a.<br>GP OS: n.a.<br><br>No CT-scan available	VA: ODS 0.1<br>Strabismus pattern: V<br>Strabismus type: exotropia<br>Refraction: hypermetropia, astigmatism<br>OD +7.50=C-2.50x165<br>OS +6.0=C-0.50x180<br>Binocular vision: not present	VA: OD 0.05, OS 0.0<br>Strabismus pattern: V<br>Strabismus type: exotropia<br>Refraction: hypermetropia, astigmatism<br>OD +7.5=C-2.50x162<br>OS +6.25=C-1.00x178<br>Binocular vision: not present<br>Torticollis: present	Improvements:<br>VA increase 0.05 OD and 0.1 OS.<br><br>Deteriorations:<br>Torticollis
53	CFNS<br>Female	10.7	Facial bipartition	Esthetical	AIOD: n.a.<br>GP OD: n.a.<br>GP OS: n.a.	AIOD: n.a.<br>GP OD: n.a.<br>GP OS: n.a.	VA: ODS 0.1<br>Strabismus pattern: V	VA: ODS 0<br>Strabismus pattern: V	Improvements:<br>VA increase 0.1 ODS.

					No CT-scan available	No CT-scan available	Strabismus type: exotropia, hypertropia<br>Refraction: hypermetropia, astigmatism<br>OD +2.00=C-3.50x180<br>OS +1.00=C-1.00x175<br>Binocular vision: poor	Strabismus type: exotropia, hypertropia<br>Refraction: hypermetropia, astigmatism<br>OD +1.50=C-3.75x170<br>OS +0.25=C-1.75x180<br>Binocular vision: moderate	Binocular vision from poor to moderate.<br><br>Deteriorations:<br>-
54	CFNS Female	10.6	Facial bipartition	Esthetical	AIOD: 35.1<br>GP OD: 15.7<br>GP OS: 14.2	AIOD: 22.0<br>GP OD:12.2<br>GP OS: 11.7	VA: OD 0.7, OS 0.4<br>Strabismus pattern: V<br>Strabismus type: esotropia<br>Refraction: hypermetropia, astigmatism<br>OD +0.50=C-1x25<br>OS +1.00=C-0.25x180<br>Binocular vision: poor	VA: OD 0.7, OS 0.5<br>Strabismus pattern: V<br>Strabismus type: esotropia<br>Refraction: astigmatism<br>OD 0.00=C-2.00x180<br>OS +0.75=C-2.00x165<br>Binocular vision: not present	Improvements:<br>Binocular vision improved 9 months postoperatively from poor to moderate after additional strabismus surgery.<br><br>Deteriorations:<br>VA 0.1 decrease OS.<br>Increase esotropia OD.<br>Astigmatism ODS.
55	CFNS Female	9.4	Facial bipartition	Esthetical	AIOD: 37.2<br>GP OD: 18.7<br>GP OS: 19.7	AIOD: 31.9<br>GP OD: 18.1<br>GP OS: 18.7	VA: OD 0.3, OS 0.15<br>Strabismus pattern: V<br>Strabismus type: exotropia<br>Refraction: hypermetropia, astigmatism<br>OD +2.50= C-0.50x75<br>OS +4.00=C-1.50x20<br>Binocular vision: not measured	VA: OD 0.2, OS 0.1<br>Strabismus pattern: V<br>Strabismus type: exotropia<br>Refraction: hypermetropia, astigmatism<br>OD +2.50=C-0.25x75<br>OS +4.75=C-1.75x12<br>Binocular vision: not measured	Improvements:<br>VA increase 0.1 OD, 0.05 OS.<br><br>Deteriorations:<br>Exotropia OD increased postoperatively, for which patient received additional strabismus surgery.
56	CFNS Female	9	Facial bipartition	Esthetical	AIOD: 37.1<br>GP OD: 18.1<br>GP OS: 19.3	AIOD: 26.8<br>GP OD: 17.0<br>GP OS: 17.9	VA: ODS 0.1<br>Strabismus pattern: V<br>Strabismus type: exotropia OS<br>Refraction: hypermetropia, astigmatism	VA: ODS 0<br>Strabismus pattern: V<br>Strabismus type: accommodative esotropia, trace exotropia with glasses OS	Improvement<br>Binocular vision from poor to moderate.<br>VA increase 0.1 ODS.

							OD +5.00=C-0.75x04<br>OS +6.26=C-2.00x51<br>Binocular vision: poor	Refraction: hypermetropia, astigmatism<br>OD +4.75=C-1.50x120<br>OS +6.25=C-2.00x35<br>Binocular vision: moderate	Deteriorations:<br>Patient had pre-operatively an intermitting exotropia OS this changed post-operatively to accommodative esotropia OS, and with glasses on a trace of exotropia OS was seen, this had no negative effect on binocular vision. Patient did not have additional strabismus surgery.
57	CFNS<br>Male	8.9	Facial bipartition	Esthetical	AIOD: n.a.<br>GP OD: n.a.<br>GP OS: n.a.<br><br>No CT-scan available	AIOD: n.a.<br>GP OD: n.a.<br>GP OS: n.a.<br><br>No CT-scan available	VA: ODS 0<br>Strabismus pattern: V<br>Strabismus type: esophoria OS<br>Refraction: hypermetropia, astigmatism<br>OD +2.50=C-0.50x172<br>OS +1.25<br>Binocular vision: moderate	VA: OD 0, OS 0.05<br>Strabismus pattern: V<br>Strabismus type: esotropia<br>Refraction: hypermetropia, astigmatism<br>OD +2.50=C-0.50x175<br>OS +1.25<br>Binocular vision: not present<br>Motility: upshoot adduction, abduction restriction<br>Torticollis: present	Improvements:<br>-<br>Deteriorations:<br>Postoperatively the present esophoria changed to esotropia OS.<br>VA decrease of 0.05 OS.<br>Torticollis.<br>Binocular vision from moderate to not present due to increase esotropia.
58	CFNS<br>Female	7.9	Facial bipartition	Esthetical	AIOD: 33.4<br>GP OD: 17.3<br>GP OS: 15.6	AIOD: 22.1<br>GP OD: 12.2<br>GP OS: 11.7	VA: OD 0.5, OS 0.7<br>Strabismus pattern: V<br>Strabismus type: esotropia<br>Refraction: hypermetropia, astigmatism	VA: ODS 0.7<br>Strabismus pattern: V<br>Strabismus type: esotropia<br>Refraction: hypermetropia, astigmatism	Improvements:<br>-<br>Deteriorations:<br>Esotropia OS.<br>VA decrease 0.2 OD.

							OD +3.0=C-2.00x23<br>OS +2.75=C-0.75x156<br>Binocular vision: poor	OD +2.75=C-2.25x15<br>OS +3.00=C-2.00x165<br>Binocular vision: poor	Diplopia (resolved within 1 month).<br>Astigmatism OS.
59	CFNS<br>Female	6.9	Facial bipartition	Esthetical	AIOD: 38.8<br>GP OD: 17.3<br>GP OS: 19.0	AIOD: n.a.<br>GP OD: n.a.<br>GP OS: n.a.<br><br>No CT-scan available.	VA: OD 0.4, OS 0.7<br>Strabismus pattern: V<br>Strabismus type: exotropia<br>Refraction: hypermetropia, astigmatism<br>OD +1.75=C-0.50x32<br>OS +3.00=C-3.75x150<br>Binocular vision: not present<br>Motility: upshoot adduction<br>Amblyopia: present ODS<br>Torticollis: present	VA: OD 0.5, OS 0.7<br>Strabismus pattern: V<br>Strabismus type: exotropia, hypotropia<br>Refraction: hypermetropia, astigmatism<br>OD +1.50<br>OS +3.00=C-4.50x145<br>Binocular vision: not present<br>Motility: upshoot adduction<br>Amblyopia: present ODS<br>Lacrimal: dryness complaints	Improvements:<br>Torticollis<br><br>Deteriorations:<br>Dryness complaints.<br>Hypotropia OS.<br>VA decrease 0.1 OD.<br><br>Explanation: VA OD worsened due to amblyopia.
60	CFNS<br>Female	6.9	Facial bipartition	Esthetical	AIOD: 35.0<br>GP OD: 16.3<br>GP OS: 16.1	AIOD: 17.5<br>GP OD: 12.3<br>GP OS: 11.9	VA: OD 0.8, OS 0<br>Strabismus pattern: V<br>Strabismus type: exotropia<br>Refraction: hypermetropia, astigmatism<br>OD +6.00=C-3.00x160<br>OS +3.75=C-1.25x2<br>Binocular vision: not measured<br>Amblyopia: present OD	VA: OD 0.5, OS 0.15<br>Strabismus pattern: V<br>Strabismus type: exotropia, hypotropia OS<br>Refraction: hypermetropia, astigmatism<br>OD +6.50=C-3.00x160<br>OS +3.75=C-1.50x177<br>Binocular vision: not measured<br>Amblyopia: present OD	Improvements:<br>VA increase 0.3 OD<br><br>Explanation: VA OD improved due to amblyopia treatment.<br><br>Deteriorations:<br>VA decrease 0.15 OS.<br>Post-operatively hypotropia OS.
61	CFNS<br>Female<br>9092324	5.2	Facial bipartition	Esthetical	AIOD: 39.1<br>GP OD: 20.8<br>GP OS: 21.1	AIOD: 24.0<br>GP OD: 14.4<br>GP OS: 14.1	VA: not correctly measured<br>Strabismus pattern: V<br>Strabismus type: esotropia<br>Refraction: hypermetropia, astigmatism<br>OD +5.00=C-3.75x10<br>OS +4.25=C-2.50x175<br>Binocular vision: not present	VA: ODS 0.6<br>Strabismus pattern: A<br>Strabismus type: esotropia<br>Refraction: hypermetropia, astigmatism<br>OD +5.5=C-3.75x7<br>OS +4.75=C-4.25x164<br>Binocular vision: poor	Improvements:<br>Binocular vision from not present to poor.<br><br>Deteriorations:<br>Pattern change.

62	CFNS<br>Female	4.9	Facial bipartition	Esthetical	AIOD: 34.5<br>GP OD: 16.1<br>GP OS: 17.4	AIOD: 27.0<br>GP OD: 14.5<br>GP OS: 14.3	VA: ODS 0.1<br>Strabismus pattern: V<br>Strabismus type: exotropia<br>Refraction: hypermetropia, astigmatism<br>OD +2.75=C-2.25x165<br>OS +3.0=C-2.25x10<br>Binocular vision: moderate	VA: ODS 0.1<br>Strabismus pattern: V<br>Strabismus type: exotropia<br>Refraction: hypermetropia, astigmatism<br>OD +3.25=C-3.00x160<br>OS +3.25=C-3.5x180<br>Binocular vision: moderate	Improvements:<br>-<br>Deteriorations:<br>-
63	CFNS<br>Female	3.7	Facial bipartition	Esthetical	AIOD: 39.7<br>GP OD: 16.6<br>GP OS: 14.6	AIOD: 33.3<br>GP OD: 16.6<br>GP OS: 14.9	VA: OD 0.6, OS 0.5<br>Strabismus pattern: V<br>Strabismus type: exotropia<br>Refraction: hypermetropia, astigmatism<br>OD +0.75=C-3.25x153<br>OS +3.00=C-3.25x31<br>Binocular vision: not measured	VA: OD 0.6, OS 0.2<br>Strabismus pattern: V<br>Strabismus type: exotropia<br>Refraction: hypermetropia, astigmatism<br>OD +1.25=C-3.75x172<br>OS +3.00=C-4.5x27<br>Binocular vision: not measured	Improvements:<br>VA increase 0.3 OS<br><br>Explanation: VA improved due to wearing glasses postoperatively.<br><br>Deteriorations:<br>-
